# Bioremediation: a genuine technology to remediate radionuclides from the environment

**DOI:** 10.1111/1751-7915.12059

**Published:** 2013-04-26

**Authors:** Dhan Prakash, Prashant Gabani, Anuj K Chandel, Zeev Ronen, Om V Singh

**Affiliations:** 1 Institute of Microbial Technology (CSIR)Sector 39-A, Chandigarh, 160036, India; 2 Department of Environmental Hydrology & Microbiology, Ben-Gurion University of the NegevSede- Boqer Campus, 84990, Israel; 3 Division of Biological and Health Sciences, University of PittsburghBradford, PA, 16701, USA; 4 Department of Biotechnology, School of Engineering of Lorena, University of Sao PauloLorena, Brazil

## Abstract

Radionuclides in the environment are a major human and environmental health concern. Like the Chernobyl disaster of 1986, the Fukushima Daiichi nuclear disaster in 2011 is once again causing damage to the environment: a large quantity of radioactive waste is being generated and dumped into the environment, and if the general population is exposed to it, may cause serious life-threatening disorders. Bioremediation has been viewed as the ecologically responsible alternative to environmentally destructive physical remediation. Microorganisms carry endogenous genetic, biochemical and physiological properties that make them ideal agents for pollutant remediation in soil and groundwater. Attempts have been made to develop native or genetically engineered (GE) microbes for the remediation of environmental contaminants including radionuclides. Microorganism-mediated bioremediation can affect the solubility, bioavailability and mobility of radionuclides. Therefore, we aim to unveil the microbial-mediated mechanisms for biotransformation of radionuclides under various environmental conditions as developing strategies for waste management of radionuclides. A discussion follows of ‘-omics’-integrated genomics and proteomics technologies, which can be used to trace the genes and proteins of interest in a given microorganism towards a cell-free bioremediation strategy.

## Introduction

In addition to the occasional disastrous accidents at nuclear facilities such as the Chernobyl disaster of 1986 and the Fukushima Daiichi nuclear disaster in 2011, the extensive use of radioactive materials at research and development, biomedical, and industrial sites has created a great accumulation of radioactive waste. Fredrickson and colleagues (2004) reported that during World War II, ∼90 million gallons of high-level radioactive waste accumulated across the USA. Most radioactive wastes are generated by nuclear power plants, which contribute ∼95% of the radioactivity generated from all sources (Ahier and Tracy, 1995; Tamponnet and Declerck, 2008). In the environment, even a small concentration of radionuclides can have an impact for a prolonged period of time due to their long half-life. As a result, the impact of radionuclide pollutants is growing with time. The commonly encountered radionuclides include cobalt-60 (^60^Co), plutonium-239 (^239^Pu), radium-226 (^226^Ra), radon-222 (^222^Rn), technetium-99 (^99^Tc), thorium-232 (^232^Th) and uranium-238 (^238^U). However, the typical radionuclides produced through nuclear reactors via the splitting of elemental atoms are thallium-201 (^201^Tl), iridium-192 (^192^Ir), caesium-137 (^137^Cs) and strontium-90 (^90^Sr), which take a significantly long time to decay (Kurnaz *et al*., 2007). Additionally, ^238^U decays to form ^226^Ra, which has a half-life of 1600 years.

Exposure to radionuclides or radiation causes acute health effects that begin with nausea, vomiting and headaches. With increased exposure a person may also experience fatigue, weakness, fever, hair loss, dizziness disorientation, diarrhoea, blood in stool, low blood pressure and ultimately death. Foetuses are particularly vulnerable to the effects of radiation at the cellular level, which can result in smaller head or brain size, poorly formed eyes, abnormal growth and mental retardation (Nussbaum, 2007; Al-Zoughool and Krewski, 2009; Bogutskaya *et al*., 2011). Studies have revealed that long-term exposure to radionuclides leads to an elevated risk of leukaemia, leucopenia, kidney damage and genetic damage, which can result in lethal health problems that can pass into the next generation (Mohner *et al*., 2006).

**Figure 1:**
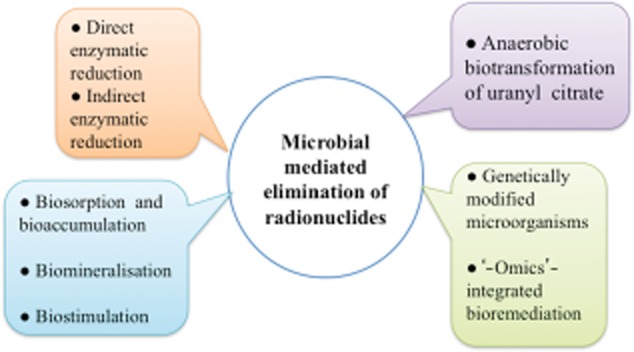
Summarization of various biotechnological approaches for bioremediation of radionuclides.

Currently, excavation and shipping to a distant waste disposal location is the most common means of eradicating soil contaminated with radionuclides. The costs of cleaning up these sites are estimated to be in excess of a trillion dollars in the USA and 50 billion pounds sterling in the UK (Lloyd and Renshaw, 2005). Given the high costs of physiochemical approaches, there has been an unprecedented interest in microbes and plants with radionuclides for decontamination of sediments and waters impacted by nuclear waste (Lloyd *et al*., 2003; Kumar *et al*., 2007). Figure 1 summarizes the various biotechnological approaches for bioremediation of radionuclides. Bioremediation via microorganisms can be an attractive alternative to excavating contaminated soil. Microorganisms such as *Rhodanobacter* sp. and *Desulfuromus aferrireducens* were observed to be able to interact with these contaminants (Amachi *et al*., 2010; Green *et al*., 2012). The interaction of site-specific microorganisms initiates solubility of transformed radionuclides by addition or removal of electrons, thus increasing the mobility of the contaminant and allowing it to be easily flushed from the environment (Amachi *et al*., 2010). This microbial-mediated biotransformation presents opportunities for bioremediation of radionuclides in the environment, either to immobilize them in place or to accelerate their removal. This article aims to interpret the procedure by which microorganisms assist in eliminating radionuclides from the environment and how they influence the toxicity and transport of radionuclides.

## Microorganisms: an asset in elimination of radionuclides

**Figure 2:**
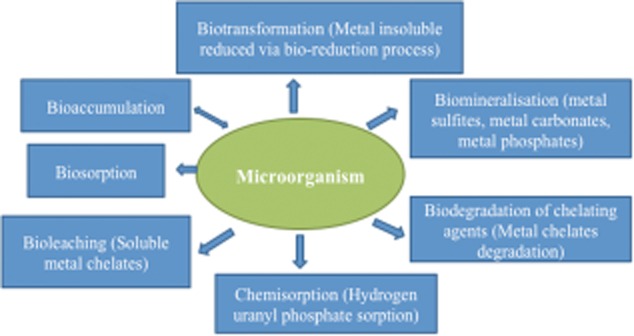
Linkage of metals with microorganism: key interaction for bioremediation.

Bioremediation of environmental niches such as soil, sediments and water contaminated with radionuclides can be achieved through biologically encoded changes in the oxidation state. Changes in speciation such as detoxification of mercury by methylation [Hg(CH_3_)_2_] can alter the solubility, transport properties and toxicity of radionuclides (Wang *et al*., 2012). The bioremedial strategies for radionuclides depend on the active metabolizing capabilities of microorganisms. Radionuclides can be solubilized by direct and indirect enzymatic reduction through oxidation–reduction, change in pH and Eh (activity of electrons), biosorption by mass, biodegradation of radionuclide–organic complexes or biosorption by biomass (Holker *et al*., 2002; Law *et al*., 2010; Hegazy and Emam, 2011). Microbial activity during the biotransformation of radionuclides is greatly influenced by electron donors and acceptors, nutrients, and environmental factors. Figure 2 shows the possible mechanistic linkages of metals with microorganism: key interaction for bioremediation.

### Direct enzymatic reduction of radionuclides

**Figure 3:**
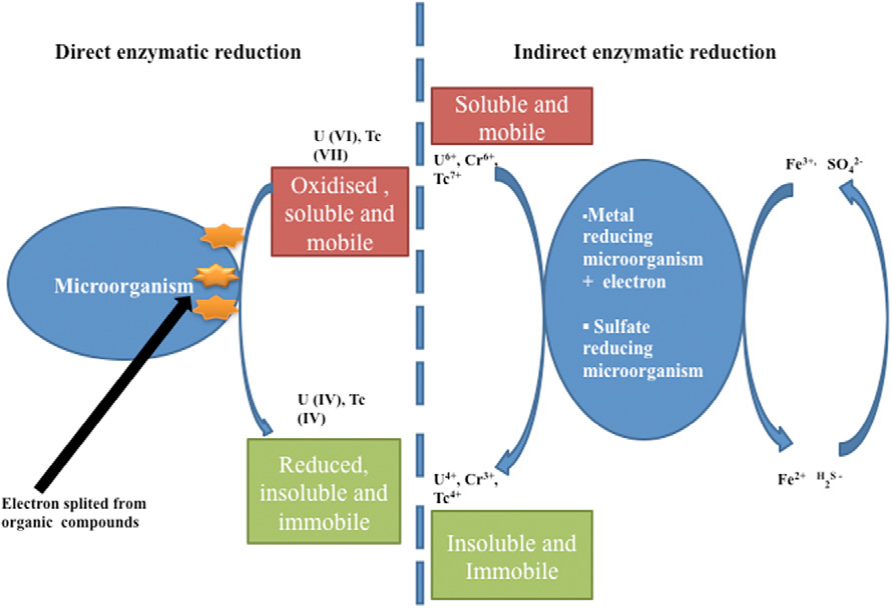
Depiction of direct enzymatic reduction and indirect mobilization of radionuclides by metal-reducing microorganisms via capturing of electrons derived by organic compounds (lactate or acetate).

The oxidized forms of radionuclides are highly soluble in aqueous medium, which makes them mobile in groundwater, whereas the reduced species are highly insoluble and often precipitate from the solution. A direct enzymatic reduction of soluble U(VI) to insoluble species is presented in Fig. 3. Wildung and colleagues (2000) reported enzymatic reduction of U(VI) on the surface of the microorganism *Shewanella putrefaciens*. A *c*-type cytochrome of mass 9.6 kDa was observed in the periplasm of *S. putrefaciens* required for U(VI) reduction. The studies showed that the mutant was unable to synthesize *c*-type cytochrome, so it did an *in vitro* reduction of U(VI). The purified tetrahaem cytochrome *c*3 protein from *Desulfovibrio vulgaris* was observed to reduce U(VI) *in vitro* using U(VI) reductase in combination with hydrogenase as its physiological electron donor (Lovley, 2003; Lovley and Phillips, 1994). *In vivo* studies showed that a homologue of *Desulfovibrio desulfuricans* (G20) confirmed the role of cytochrome *c*3 in hydrogen-dependent U(VI) reduction. Similarly, Lloyd and colleagues (2003) identified a homologous cytochrome (PpcA), a trihaem periplasmic cytochrome *c*7 of the Fe(III)-reducing bacterium *Geobacter sulfurreducens* that may also play a role in U(VI) reduction *in vitro*.

^99^Tc, another long-lived radionuclide (half-life 2.13 × 10^5^ years) that mostly occurs in nuclear wastes, has attracted considerable interest (Tamponnet and Declerck, 2008; Alliot *et al*., 2009). Tc(VII) has feeble ligand-complexing capabilities and is difficult to remove from solution using conventional chemical methods. Metal-reducing microorganisms can reduce Tc(VII) and precipitate the radionuclide into low-valency oxide TC(IV). The studies demonstrated reduction of Tc solubility (Henrot, 1989; Pignolet *et al*., 1989). However, the direct microbial enzymatic reduction of Tc(VII) was first observed by Lloyd and Macaskie (1996) using *S. putrefaciens* and *Geobacter metallireducens*. The reduction of Tc(VII) was quantified using a phosphorimager technique. In another study, X-ray absorption spectroscopy showed Tc(IV) as the final insoluble oxidation state after Tc(VII) was reduced by *G. sulfurreducens* and *S. putrefaciens* (Wildung *et al*., 2000). It is well reported that Tc(VII) reduction has produced fortuitous biochemical side reactions in the organisms studied to date.

Further, the biochemical basis of Tc(VII) reduction has been studied in *Escherichia coli*. Initial studies demonstrated that anaerobic cultures of *E*. *coli* reduced Tc(VII) with the reduced radionuclide precipitated within the cell (Lloyd *et al*., 1997). Results obtained with wild-types and mutants defective in the synthesis of regulatory or electron transfer proteins were used to construct a model for Tc(VII) reduction by *E*. *coli*. The model demonstrates that three major components of formate hydrogenlyase catalyse the transfer of electrons from dihydrogen to Tc(VII). However, according to the model, the formate dehydrogenase component is required only if formate is supplied as an electron donor for Tc(VII) reduction in place of hydrogen. The model was authenticated by a mutant strain unable to synthesize all three components of hydrogenase to reduce Tc(VII) when either hydrogen or formate was supplied as an electron donor (Lloyd *et al*., 1997). The identification of three components of formate hydrogenlyase and Tc(VII) reductase of *E*. *coli* opened up a new avenue to screen for organisms with naturally enhanced ability to act against Tc(VII).

Several other organisms are known to have naturally high formate hydrogenlyase activity. In addition, the uptake of hydrogenase was established to couple the oxidation of formate or hydrogen to Tc(VII) reduction (Lloyd *et al*., 2001). *Desulfovibrio desulfuricans* and related organisms were observed to utilize formate as an efficient electron donor for Tc(VII) reduction (Lloyd *et al*., 1999). This observation is consistent with the existence of a rudimentary formate hydrogenlyase (FHL) complex located in the periplasm of microorganisms (Peck, 1993). These studies have also been confirmed by the role of periplasmic Ni-Fe hydrogenase in Tc(VII) reduction by a relative in the N subclass of *Desulfovibrio fructosovorans* (De Luca *et al*., 2001). Subsequent studies on the development of a bioprocess to decontaminate Tc(VII)-contaminated water have focused on the use of immobilized cells of sulfate-reducing bacteria such as *D. desulfuricans,* which are capable of treating low concentrations of Tc(VII) against a high concentration of nitrate ions, which is frequently found in nuclear waste (Lloyd *et al*., 1999). Fujimoto and Morita (2006) reported a novel strain of Halomonas (Tc-202), isolated from a marine environment, that was capable of removing Tc(VII) from solid- and aqueous-phase material aerobically.

Although U and Tc remain the highest-priority radionuclide pollutants in most medium- and low-level radioactive wastes, other actinides including Th, Np, Pu and Am also occur at the contaminated sites (Lloyd and Macaskie, 2000; Tamponnet and Declerck, 2008). Iron-reducing bacteria such as *Geobacter* sp. and *Rhodoferax ferrireducens* have the metabolic potential to reduce these radionuclides enzymatically (Kim *et al*., 2012). These findings are significant, as most tetravalent actinides are acquiescent to bioremediation due to their high ligand-complexing abilities. In addition, these actinides could be immobilized in sediments containing active biomass (Peretrukhin *et al*., 1996). Although it is possible for Fe(III)-reducing bacteria to reduce and precipitate actinides in one step [e.g. soluble U(VI) to insoluble U(IV)], few studies support the direct formation of an insoluble mineral stage; rather they indicate the formation of a cation prone to bioprecipitation (Lloyd *et al*., 2000). This phenomenon is illustrated when considering highly soluble Np(V), which was reduced to insoluble Np(IV) by *S. putrefaciens*, then removed as an insoluble phosphate biomineral by a phosphate-liberating *Citrobacter* sp. Lloyd and colleagues (2000) studies have also suggested that the reduction of Pu(IV) to Pu(III) can be achieved by Fe(III)-reducing bacteria and Pu(III) was reported to reoxidize spontaneously (Rusin *et al*., 1994).

### Indirect enzymatic reduction of radionuclides

The enzymatic bioreduction of radionuclides can be triggered through indirect reduction of soluble contaminants in sedimentary and subsurface environments by metal-reducing or sulfate-reducing microorganisms. One approach is to couple the oxidation of organic compounds or hydrogen to the reduction of iron Fe(III) or sulfur S(IV) in the form of sulfate. The iron Fe(III) can be bioreduced into Fe(II) and S(VI) into S(II) (hydrogen sulfide, H_2_S). The product can be further reduced chemically to yield separate or multi-component insoluble species (van Hullebusch *et al*., 2005). The reduced forms of these metals are insoluble and can precipitate as either reduced oxide or hydroxide minerals. Similarly, sulfate-reducing bacteria may be stimulated to produce hydrogen sulfide. Several microorganisms such as *Microbacterium flavescens* grown in the presence of other radionuclides (i.e. U, Th, Am and Pu) produced unidentified compounds such as organic acids, siderophores and extracellular metabolites capable of dissolving and mobilizing radionuclides into the soil. These compounds may also help to transport radionuclides within the cells (John *et al*., 2001). Figure 3 depicts the direct enzymatic reduction and indirect mobilization of radionuclides by metal-reducing microorganisms via capturing of electrons derived by organic compounds (lactate or acetate).

Pu(IV), Th(IV), U(VI) and Fe(III) have chemical and biochemical similarities because of the ubiquity of siderophore-producing microbes. The iron-sequestering agents produced by such microorganisms are crucial to increase the solubility and bioavailability of radionuclides. Premuzic and colleagues (1985) reported the production of extracellular chelating agents in *Pseudomonas aeruginosa* that can bioaccumulate uranium. This report prompted several chelating agents for thorium and uranium when grown with these metals. Brainard and colleagues (1992) solubilized hydrous PuO_2_(s) using the siderophores enterobactin, desferrioxamin, carboxylate amino polycarboxylate and catecholate ligands. The report conclusively showed that enterobactin siderophores are extremely effective in solubilizing actinide oxides of plutonium, among the tested other chelators.

Several microorganisms produced extracellular complexes in the presence of Pu and Th that increased the concentration of Pu and Th in soil-column elutes compared with controls. The increased mobility of Pu and Th in soil resulted from the formation of neutral and negatively charged Pu and Th complexes. In the presence of known microbial metabolites such as synthetic ligand [ethylenediamine tetraacetic acid (EDTA)] and citric acid, Pu(VI) and Th(IV) were reduced to Pu(IV) and Th(IV) respectively before complex formation, suggesting that the latter valence state would be the dominant one associated with the organic complexes in soil (Panak and Nitsche, 2001).

## Biosorption and bioaccumulation

Biosorption is the sequestration of positively charged metal ions to the negatively charged cell membranes and polysaccharides secreted on the outer surfaces of bacteria through slime and capsule formation. Sorption of metals to intact cells is directed by a multiplicity of mechanisms and interactions that are not yet fully understood. Langley and Beveridge (1999) described the role of carboxyls in the binding of metal cations to *O*-side-chains of lipopolysaccharide (LPS) and concluded that metal was most likely to bind to phosphoryl groups in the core-lipid ‘A’ of LPS, and the negatively charged side-chains affected cell hydrophobicity in Gram-negative bacteria. Similarly, Khani and colleagues (2005) described the effective adsorption of radionuclide U(VI) by the brown marine alga *Cystoseira indica* and observed that the pre-treatment of the alga with calcium could enhance the adsorption efficiency of several radionuclides. Several microorganisms including *Citrobacter freudii* and *Firmicutes* have been reported as radionuclide biosorbents (Haferburg *et al*., 2007; N'Guessan *et al*., 2008; Xie *et al*., 2008).

Sorption of metals plays a key role in microbe-metal interactions. The precipitation of uranyl phosphate by *Citrobacter* is initiated by interaction (electrostatic) with phosphate groups in the LPS. This interaction provides nucleation sites for mineral formation and protects an outer membrane phosphatase (Macaskie *et al*., 2000). Panak and colleagues (2002) examined the sorption of Pu(VI) with bacterial resting cells and concluded that the interaction with bacteria caused changes in the oxidation state of Pu(V) due to endogenous respiration. Extended X-ray Absorption Fine Structure (EXAFS) analysis of Pu associated with the cells showed that Pu(VI) was primarily bound to the phosphate groups on the cell surface; the biosorption efficiency looked to be positively related to temperature and could occur within hours. This process is substantially faster than direct bioreduction. However, radionuclide-contaminated ground is normally poor in biomass concentration due to the higher toxicity of radionuclides. Therefore, biosorption alone may not be sufficient to bioremediate radionuclides unless the ground biomass content is increased. To improve the efficiency of biosorption, elegant strategies such as recombinant DNA technology and stimulated growth of microbes in the contaminated ground could enhance radionuclide remediation.

### Biostimulation

Biostimulation using specific communities of microorganisms is another mechanism to enhance the bioremediation of radionuclides. An *in situ* remediation method was developed to reduce the bioavailability and prevent the further spread of uranium in groundwater by promoting the activity of dissimilatory sulfate- and iron-reducing organisms (Vrionis *et al*., 2005). In biostimulation, nitrate serves as an energetically favourable electron acceptor for metal-reducing bacteria in nitric acid co-contaminated sediments (DiChristina, 1992). A lack of microbial reduction in U(VI) has been reported if the sediment is co-contaminated with nitrate (Finneran *et al*., 2002). Sakadevan and colleagues (1999) reported that nitrate reduction was inhibited due to the presence of several heavy metals. Therefore, an attempt was made to resolve this issue by the *ex situ* treatment and removal of heavy metals and nitrate prior to *in situ* biostimulation to reduce the U(VI) (Wu *et al*., 2006).

At sites co-contaminated with metallic ions beyond toxic levels, the microbial resistance of endogenous microbial populations is critical for *in situ* biostimulation. A number of microbes has been shown to carry out reductive precipitation of radionuclides (e.g. *Desulfovibrio* sp., *Geobacter* sp. and *Shewanella* sp.); however, the resistance of these organisms to non-reducible heavy metals could possibly limit their *in situ* activity. Martinez and colleagues (2006) reported the presence of heavy-metal-resistance genes within the endogenous microbial community at the Oak Ridge Field Research Center (FRC) site. In the presence of heavy metals, the potential for biostimulation was improved by using ethanol for bioreduction of nitrate, followed by the successful reduction and *in situ* immobilization of U(VI) (Istok *et al*., 2004; Nyman *et al*., 2006). Therefore, the addition of a suitable carbon source was recommended to stimulate radionuclide bioreduction at co-contaminated sites.

Another novel method was developed to bioremediate areas contaminated with high concentrations of uranium (> 1000 μm) at low pH and high nitric acid concentrations (Wu *et al*., 2006). The method was based on raising pH with the addition of carbon (ethanol) to stimulate the growth of denitrifying and radionuclide-reducing bacteria, using a hydraulic recirculation system composed of an outer and inner loop. An *ex situ* system was also developed to prevent clogging of the hydraulic recirculation system with nitrogen gas, aluminium, calcium precipitation and biomass due to denitrification. After 1 year at optimized conditions, the concentrations of U(VI) and nitrate had been reduced to 5 μm and 0.5 μm respectively. After 2 years of preconditioning, the level of U(VI) was reduced to 0.126 μm – below the EPA's recommended level. These studies suggested that alternation of geochemical and hydrological parameters can effectively biostimulate radionuclide-reducing bacteria *in situ*. Remediation of high U(VI) concentrations with ethanol-biostimulated biofilm is an alternative approach that can effectively reduce 87% of U(VI) (Marsili *et al*., 2007).

## Biomineralization of radionuclides

Microorganisms can interact with metal ions and immobilize them to transformation. Some microorganisms generate biofilms to bind significant quantities of metallic ions, which can serve as a platform for the precipitation of insoluble minerals. Microorganism *Citrobacter* sp. was reported to be able to produce deposits of metal phosphate enzymatically. Polycrystalline NaUO_2_Po_4_ accumulates in and around the cell wall of *Citrobacter* by sorption to LPS and the activity of an outer membrane acid-phosphatase (Keasling *et al*., 2000). The mineral formation is driven by two gradients in the outer membranes: an incoming UO_4_ and an outgoing PO_4_, resulting in total removal of U from the solution and binding of 1 mg NaUO_2_Po_4_ per mg of the cell. Keasling and colleagues (2000) prepared a recombinant version of this mechanism by cloning the gene encoding polyphosphate kinase in *P. aeruginosa,* resulting in the precipitation of a complex containing both phosphorous and uranium on the cell surface.

### Biomineralization via microbial-generated ligands

Chelating agents are present in wastes because they are widely used for the decontamination of nuclear reactors and equipment, cleanup operations, and the separation of radionuclides. Several organic agents, such as citric acid, hydroxyl-acetic acid, oxalic acid, tartaric acid, EDTA, diethylenetriamine pentaacetic acid (DTPA), nitrilotriacetic acid (NTA) and *N*-hydroxyethylenediamine triacetic acid (HEDTA) have been used to complex with radionuclides. These metal chelates undergo aerobic or anaerobic biodegradation and cause the precipitation of released ions as water-insoluble hydroxides or oxides, thus retarding their migration into groundwater. Citrate has been found useful as a chelating agent in decontamination because it forms highly soluble metal–citrate complexes that can be degraded by microorganisms, resulting in subsequent re-precipitation of metals. Stable complexes such as bidentate, tridentate and polynuclear complexes can be formed with citrate and radionuclides. Francis (1998) reported that bioidentate uranium complexed with citric acid was readily biodegraded, whereas tridentate was recalcitrant.

### Anaerobic and aerobic biotransformation of uranyl–citrate

Sulfate-reducing *D. desulfuricans* and facultative iron-reducing *Schwanella alga* reduced U(VI) complexes with citrate to U(IV) anaerobically, but little uranium was precipitated (Ganesh *et al*., 1997). Similarly, *Clostridium* sp. (ATCC 53464) metabolized glucose but not citrate and reduced U(VI)–citrate into U(IV)–citrate only in the presence of glucose. Another *Clostridium sphenoides* (ATCC 19403) metabolized bidentate Fe(III)–citrate complex. This bacterium reduced Fe(III) to Fe(II) and concomitantly metabolized citric acid. In contrast, U(VI)–citrate was reduced to U(IV)–citrate by the bacterium in the presence of electron donor glucose or uncomplex citric acid. Therefore, these results indicate that the complex of uranium with citric acid is readily available for organisms as an electron acceptor, despite their incapability to metabolize organic ligands of radionuclides.

Some organisms metabolize citric acid aerobically using the enzymes aconitase and citrate lyase. The enzyme aconitase isomerizes citrate into isocitrate in the TCA cycle. However, citrate lyase drives the anaerobic metabolism of citric acid. Francis (1998) tested the ability of *Acinetobacter, Citrobacter* and *Pseudomonas* from low-level radioactive waste sites to metabolize citric acid into a uranyl–citrate complex. These cultures failed to metabolize a stable binuclear complex of uranyl–citrate, possibly because the uranyl–citrate complex did not transport inside the cells, as revealed by ^14^C labelling analysis. Furthermore, a cell-free extract of the tested organism showed that the binuclear U–citrate complex was completely degraded. Francis and colleagues (2002) tested the speciation of uranium and citric acid as a function of pH and concluded that the amount of U–citrate complex decreased rapidly above pH 6.0, whereas free citric acid was not biodegraded and transported.

Similarly, citric acid can form ternary mixed-metal complexes with a number of metals, and the complex formed affects the biodegradation and mobility of metal–citrate complexes (Dodge *et al*., 2002). For example, the biotransformation of Fe–U–citrate complex was recalcitrant. When onefold excess citric acid was added to the 1:1:2 Fe–U–citrate complex, the excess citric acid was completely degraded. However, with twofold excess citric acid, 1:1:1 Fe–U-citric acid remained in solution after the excess citric acid was biodegraded. Therefore, this study suggested that Fe–U–mixed-metal citric acid complexes resist biodegradation and may persist in the environment, which is a limitation of current technology and a challenge to the microbial-mediated bioremediation of radionuclides (Dodge *et al*., 2002).

### Genetically modified microorganisms

Genetic engineering (GE) and recombinant DNA technology have been employed to generate character-specific microorganisms for efficient removal of metal by sorption. Different protein constructs have been generated in which the bacterial cell surface is equipped with metal-binding polypeptides by fusion-binding domains to outer-membrane-anchored proteins that include metallothioneins (Valls *et al*., 2000a), randomly generated polypeptides (Schembri *et al*., 1999), polyhistidines and synthetic phytochelatines (Schembri *et al*., 1999; Bae *et al*., 2000). These protein constructs proved an increase in metal binding. The metallothioneins were also tested in microcosm field study (Valls *et al*., 2000b). Another approach attempted to enhance the metal accumulation by combining the specific metal transporter with MTs in the cytoplasm (Wolfram and Bauerfeind, 2009). A recombinant strain of *E*. *coli* was generated with five times the sorption ability for U(VI) radionuclides by altering the transporter genes *nixA* (*Helicobacter pylori*) and *merTP* (*Serratia marcescens*) respectively (Beckwith *et al*., 2001). Therefore, the expression of both metal transporter proteins and metal-binding peptides may enhance a strain's ability to accumulate metal ions.

The microorganism *Deinococcus radiodurans* has been studied to detoxify Cr(VI), U(VI) and Tc(VII) from soil (Fredrickson *et al*., 2000). A genetically engineered *D. radiodurans* strain was created by cloning the *E. coli* gene (*merA*) that provides the ability to utilize carbon and energy from catabolism of toluene and mercury (radioactive contaminants) (Brim *et al*., 2000). Progress has been made in constructing strains of *D. radiodurans* for radionuclide remediation; however, an *in situ* bioremediation strategy has yet to be discovered. The microorganisms *Deinococcus murrayi* and *Deinococcus geothermalis* were characterized as growing at a higher temperature range (55°C), and showing remarkable resistance against chronic irradiation (50 Gy h^−1^) (Brim *et al*., 2003).

The microbial family *Geobacteriaceae* has shown potential for radioactive metal reduction (Lloyd *et al*., 2003). The gene *dcuB* from *G. sulfurreducens,* which encodes a fumarate transporter, was engineered in *G. metallireducens* to grow with fumarate as a terminal electron acceptor, revealing the ruling approach of GE with expanded respiratory capabilities (Butler *et al*., 2006). Undoubtedly, GE microbes show promise, but their implementation for *in situ* bioremediation will require additional steps to develop safe routes of environmental cleanup.

## ‘-Omics’-implemented radionuclide bioremediation

The genome of an organism comprises its entire set of hereditary information that is converted to mRNA (the transcriptome) for protein translation. The proteome of an organism is the entire set of proteins, including enzymes, that are expressed in the organism under specific environmental conditions. In order to identify genes, proteins and enzymes involved in the bioremediation of radionuclides, it is important to study the structural and functional interactions between proteins and other metabolites. Potential genes and proteins involved in the metabolism of radionuclides can be identified and studied via advanced genomics and proteomics techniques (Nagaraj and Singh, 2010; Singh *et al*., 2011). Recent advances in next-generation sequencing, genomics and proteomics allow the expression of required proteins and enzymes of interest into radionuclide-resistant organisms for bioremediation. Further, genome-wide transcriptome analysis can provide us with a better understanding of the metabolic pathways and the physiology of the microorganisms.

The genome sequences of many microorganisms are now available and can be used for genome organization, including comparisons through microarrays (Ishii *et al*., 2007). Among several genome-wide studies of genes and proteins involved in radionuclide reduction pathways, Methe and colleagues (2003) reported that *G. sulfurreducens* had more than 100 *c*-type cytochrome gene coding regions in its genome and many of its translated proteins were involved with radionuclide reduction pathways. DNA-microarray-mediated analysis revealed 121 genes found to be upregulated in *Shewanella oneidensis* during U(VI) reduction, compared with Cr(VI) reduction, where only 83 genes were overexpressed (Bencheikh-Latmani *et al*., 2005). In a comparative genomics study, the organism *Thermococcus gammatolerans* was observed to be radioresistant among *Archaea*, expressing thioredoxin reductase (tgo180), a glutaredoxin-like protein (tg1302) and two peroxiredoxins (tg1253 and tg1220), which allowed the organism to cope with the stress of radionuclides (Zivanovic *et al*., 2009). It was found that the expression of the *NiCoT* gene in *Rhodopseudomonas palustris* CGA009 and *Novosphingobium aromaticivorans* F-199 was highly increased when the organisms were grown in the presence of radioactive cobalt (Raghu *et al*., 2008).

In addition to genomics technology, proteomics technology has been proven effective in studying proteins involved in radionuclide bioremediation. Tian and colleagues (2010) found a total of 552 differentially regulated proteins, including a cytochrome *bd* ubiquinol oxidase, on the membrane of *D. geothermalis* that were involved in the radioresistance of the organism. Many of these proteins comprised function groups including nutrient transport and metabolism, energy production and conversion, and cell wall/membrane biogenesis (Tian *et al*., 2010). In another study, cascades of enzymatic proteins in the outer membrane of *S. oneidensis* were found to effectively reduce the levels of radionuclides such as uranium and chromium (Marshall *et al*., 2006). In a well-known radioresistant bacterium, *D. radiodurans*, 2DE and MALDI-TOF MS revealed that 31 radiation-responsive proteins were significantly upregulated, including RecA and PprA, which are well known for DNA replication and repair (Lu *et al*., 2009). Other proteins included those involved in the stress response, energy metabolism, transcriptional regulation, protein turnover and chaperoning (Lu *et al*., 2009).

Understanding specific genes and their protein products is essential to understand the pathways involved in the bioremediation of radionuclides. Combining ‘-omics’-based approaches may assist in the identification of specific microbes effective for *in situ* bioremediation of radionuclides. Table 1 summarizes the major transcripts and proteins investigated in various radionuclides remediation studies.

**Table 1:** Major transcripts and their sources for radionuclides remediation

Radionuclides	Organisms	Gene/protein	Function	Reference
Uranium	*Deinococcus radiodurans*	DrPhoN	Surface-associated precipitation of uranium (5.7 g uranium g^−1^ biomass)	Misra *et al*. (2012)
Cobalt	*Rhodopseudomonas palustris* CGA0009	NiCoT	85% removal of Cobalt was achieved in a two-cycle treatment with recombinant *E. coli*	Raghu *et al*. (2008)
Cobalt	*Novosphingobium aromaticivorans* F-199	NiCoT		Raghu *et al*. (2008)
Uranium	*Sphingomonas* sp. BSAR-1	phoK	Recombinant *E. coli* precipitated > 90% of 0.5–5 mM of uranyl carbonate in less than 2 h	Nilgiriwala *et al*. (2008)
Chromate, uranyl	*Escherichia coli*	ChrR6	Expressed chromate reductase activity in addition to convert soluble U(VI) to insoluble with U(IV) with Vmax of 8,812 ± 611	Barak *et al*. (2006)
Uranium	*Salmonella enterica serovar Typhi*	phoN	Recombinant strain precipitated over 90% of the uranium from a 0.8 mM uranyl nitrate solution in 6 h	Appukuttan *et al*. (2006)
Mercury	*Escherichia coli* BL308	*merA*	Biotransformation of Hg (II) to the less toxic volatile Hg (I)	Brim *et al*. (2003)
Cadminum, zinc, cobalt	*Ralstonia eutrophus* CH34	*czc*		Diels *et al*. (1995)
Fumarate, nitrate, dimethyl sulfoxide, trimethylamine *N*-oxide (TMAO), nitrite and insoluble iron and manganese oxides	*Shewanella oneidensis*	*fccA*	Periplasmic flavocytochrome *c* fumarate reductase	Saffarini *et al*. (2003)
Uranium	*Desulfovibrio vulgaris*	*ctyc3*	Reduce uranium(VI) to uranium(IV) with hydrogen as the electron donor	Payne *et al*. (2002)
Fe(III)	*Geobacter sulfurreducens*	PpcA	Restoration of Fe(III) reduction with acetate	Lloyd *et al*. (2003)

## Challenges, limitations and 5-year view

Despite the progress that has been made in the field of radionuclide bioremediation using microorganisms, many challenges lie ahead. A basic concern is to optimize the conditions and procedures for sustained and effective bioremediation in the presence of competing anions, toxic metals, organic compounds and chelating agents. Another aspect that is of interest is the reoxidation and remobilization of reduced radionuclides by microbial metabolism and abiotic mechanisms. Careful consideration needs to be given to *in situ* biostimulation or bioaugmentation where various additives (microbes or chemical ingredients) are employed to enhance microbial activity; such additives might be disruptive to natural microbiota. It is still an issue to enhance the metabolic activity by maintaining the required growth conditions, such as pH, temperature, and levels of contaminants and nutrients, including diverse chemical parameters of selective microbial populations for *in situ* bioremediation. In addition, it is necessary to recognize the heterogeneous nature of contaminants on sites that can lead to an uneven flow of the liquid or gas containing the microbes. Due to the slow speed of achieving acceptable levels of decontamination at diverse sites, it is difficult to predict the performance of bench-scale bioremediation in field operations.

Genetically modified microorganisms have limitations in terms of liability and ethical issues from regulatory agencies such as the United States Environmental Protection Agency (USEPA). The release of GE organisms is a tedious task that requires site dependence compatibility and comparability with other organisms on site. After remediation, removal of GE microorganisms is also not an easy task. The direct immobilization of enzymes on support material could be useful for bioremediation, but it requires abundant optimization of site-specific substrates.

Recent developments in ‘-omics’-based technologies, i.e. genomics, transcriptomics and proteomics, are set to revolutionize many aspects of the biological sciences. A whole-genome transcription profile of *E. coli* has already uncovered potential strategies for metal detoxification (Brocklehurst and Morby, 2000). Because of the current availability of complete genome sequences of many radioresistant microorganisms, this approach will prove useful for determining the precise mechanisms of biologically relevant radionuclide–microbe interactions. However, these discoveries in environmental science are still in their infancy stage and have yet to prove their usefulness in environmental cleanup. In the future, coordinated multidisciplinary efforts need to be made to understand the overall process by which microorganisms degrade radionuclides as well as to implement the conditions that most effectively degrade the radionuclides. Focused research exploring the necessary genes and proteins of the microbial metabolic pathway towards cell-free bioremediation will pave the way to refine these bioremediation technologies before successful field practice.

## Conclusion

Microbial transformations of radionuclides, heavy metals and minerals are a vital part of natural biosphere processes and can have beneficial consequences for the human community. As we know, the interactions between microorganisms and radionuclides are far from simple, and it is not easy to understand the wide range of environments these organisms inhabit. Study of the molecular mechanisms behind the microbial transformation of radionuclides using ‘-omics’-based approaches and exploiting them in applications such as bioremediation would assist in tracking the responsible microbial metabolic products towards cell-free bioremediation and further assist in efficient removal of radionuclides from the environment.
